# Transparency of Mandatory Information Disclosure and Concerns of Health Services Providers and Consumers

**DOI:** 10.3390/ijerph14010053

**Published:** 2017-01-09

**Authors:** Yu-Hua Yan, Chih-Ming Kung, Shih-Chieh Fang, Yi Chen

**Affiliations:** 1 Department of Medical Research, Tainan Municipal Hospital, No. 670, Chung Te Road, Tainan City 701, Taiwan; 2d0003@mail.tmh.org.tw; 2 Department of Information Technology and Communication, Shih Chien University Kaohsiung Campus, No. 200 University Road, Neimen, Kaohsiung 84550, Taiwan; alex@mail.kh.usc.edu.tw; 3 Department of Business Administration, Institute of International Business, National Cheng Kung University, No. 1, University Road, Tainan City 701, Taiwan; fangsc@mail.ncku.edu.tw; 4 Department of Superintendent’s Office, Tainan Municipal Hospital, No. 670, Chung Te Road, Tainan City 701, Taiwan

**Keywords:** mandatory information disclosure, healthcare quality disclosure, financial statement disclosure

## Abstract

Background: This study analyzed differences between transparency of information disclosure and related demands from the health service consumer’s perspective. It also compared how health service providers and consumers are associated by different levels of mandatory information disclosure. Methods: We obtained our research data using a questionnaire survey (health services providers, *n* = 201; health service consumers, *n* = 384). Results: Health service consumers do not have major concerns regarding mandatory information disclosure. However, they are concerned about complaint channels and settlement results, results of patient satisfaction surveys, and disclosure of hospital financial statements (*p* < 0.001). We identified significant differences in health service providers’ and consumers’ awareness regarding the transparency of information disclosure (*p* < 0.001). Conclusions: It may not be possible for outsiders to properly interpret the information provided by hospitals. Thus, when a hospital discloses information, it is necessary for the government to consider the information’s applicability. Toward improving medical expertise and information asymmetry, the government has to reduce the burden among health service consumers in dealing with this information, and it has to use the information effectively.

## 1. Introduction

Transparent information disclosure is intended to alleviate the conflicts of interest and negative economic consequences caused by information asymmetry [1,2]. Since national health insurance was launched in Taiwan in 1995, a sound social safety network has been established, which is dedicated to providing national medical care [3]. Domestic and foreign economic changes and increasing public expectations have resulted in increasing demand for medical treatment and financial imbalance in national health insurance. As a result, the government was forced to comprehensively implement the Global Budget Payment System in 2002 [4,5,6].

In consequence, health service providers undertook various response actions to achieve target income levels [7,8,9]. Following developments over the previous 18 years, national health insurance (NHI) underwent privatization in 1997 and faced rising premiums and co-payment issues. Such problems as inadequate social participation and insufficient information disclosure have gradually become the concern of all health service sectors. Accordingly, full-scale health insurance reforms with a macro perspective were gradually introduced, and a bill of amendment—the Second Generation NHI—was instituted. The Second Generation NHI Act was officially enforced in 2013 [10]. The present study aimed to examine the performance and concerns of health service providers and consumers in Taiwan with respect to mandatory information disclosure. The results of this research may serve as a reference for policy enforcers when introducing new policies and amending laws.

Transparent information disclosure may reduce agency problems arising from information asymmetry regarding maintaining client interests and rights. A well-founded, transparent information disclosure system enables agents to disclose financial details fully and transmit important information toward helping stakeholders make sound judgments based on sufficient data [11,12,13,14]. By contrast, without such a disclosure system, agents are likely to lead clients to misjudgments (based on incorrect information through manipulating the type, quality, and quantity of disclosed information), thereby affecting clients’ behavior and resulting in some discrepancy between laws and regulations and actual circumstances. The situations in different types of organizations also vary depending on the stakeholders’ characteristics and legal environment [15,16]. For example, information disclosure has always been the easiest way to provide stakeholders with access to information regarding hospital governance. When the transparency of information disclosure is low, there is a greater chance of hidden malpractices or problems with a hospital’s operation. Therefore, enhancing transparency is a powerful driving force in improving the governance of a hospital [17].

Generally, information disclosure includes voluntary and mandatory disclosure, the latter being enforced by laws and regulations [18]. The different aspects of information disclosure have different implications [19,20,21]. The concept of mandatory information disclosure policy is created on the premise that institutions disclose information and that stakeholders may urge the institutions to improve the content disclosed [22]. During the enforcement period of the First Generation NHI, there were no compulsory requirements about hospital information disclosure. Since 2002, the Taiwan Health Care Reform Foundation and some consumer protection groups have strongly demanded that the government publicize financial information related to large-scale hospitals. In 2004, that foundation and associated groups stated that under the circumstance of all hospitals being NHI-contracted hospitals, if hospitals refused to publicize their financial information, insurers would face difficulties in making a reasonable allocation of total medical expenses based on hospitals’ overview of their operations. It would also be impossible to effectively supervise expenditure in NHI medical expenses [23]. Transparent information disclosure not only communicates operating results and allocation of medical treatment resources; it also provides critical information for the reference of hospital management, government departments, and interested parties. Further, transparent information disclosure may be quoted as a data source in some studies and surveys and form the basis for allocating medical treatment resources; this effect cannot be disregarded.

Thus, information disclosure is the core concept of the Second Generation NHI amendment. The amendment states that all information related to policy making, coverage, quality of medical care, and service resources should be disclosed [10]. The contents of disclosure include information about the following: meetings related to important NHI motions (Articles 5, 41, and 61); participating representatives’ interest disclosures (Article 41); medical treatment technology assessment results (Article 41); insured bed ratio and number of insured beds in each NHI-contracted hospital (Article 67); financial reports of NHI-contracted medical institutions that receive specific medical expenses in the current year (Article 73); medical treatment quality of NHI-contracted medical institutions (Article 74); and material violations (Article 81). Generally speaking, enterprises choose information disclosure through three major motives: reduction in funding costs because of diminished information asymmetry [24,25,26]; resolution of proxy conflicts [2]; and compliance with the competent authority’s requirement for reduced political costs [27,28,29].

When seeking medical treatment, people in Taiwan are considered to enjoy the most lenient thresholds and restrictions in the world [5]. The NHI is not an expensive medical service, which it was originally intended to be, but operates somewhat like a 24 h convenience store. Based on the hypothesis of behavior under agency theory, the principal and agent are both rational individuals; they possess such characteristics as being self-serving, having bounded rationality and risk aversion, and being dedicated to pursuing maximized interest for themselves; the inconsistency—if any—in the goals they pursue results in the agency problem [11,12,13]. Among agency problems, information asymmetry is a primary source for generating agency cost. Therefore, upgrading the level of information disclosure will be helpful in attenuating information asymmetry between the agent and principal, thereby reducing agency cost [11,12,13]. Moreover, stakeholders will push the agency to force enterprises to improve the target content and quality of information disclosed [30]. Agency theory and system theory apparently provide important theoretical bases for institutions’ information disclosure and are an important issue about how external forces affect an institution’s behavior [30,31,32,33]. In the light of these theories, mandatory information disclosure offers important study matter.

Subject to different properties, the implications of awareness and concern through information disclosure vary [19,20,21]. Awareness highlights that individuals share common concepts and judgments with others in a social network system [34]: individuals are pressured by external force to comprehend and memorize issues in the process of awareness and judgment; in that process, they develop different judgment guidelines and adopt different values and intentional actions with the knowledge and experience they accumulate [35,36]. The awareness and concern of health service consumers toward policies will vary depending on such factors as demand, value, and interest [37,38,39]. The quality of information disclosure has become a significant concern in all health sectors in Taiwan. To improve the quality of hospital information disclosure and reduce the gap between hospitals and stakeholders, the government enacted the laws and regulations of the Second Generation NHI. To analyze the influence of health service consumers on hospital information disclosure and based on the information disclosure embodied in the NHI Act, the present study adopted an evidence-based approach to analyze transparent information disclosure in terms of the following categories: disclosure of medical management; disclosure of medical treatment; disclosure of medical quality; disclosure of financial statements; disclosure of information about violations; and disclosure of insured bed ratio. We considered it important to undertake an analysis of information disclosure under the Second Generation NHI at the time of introduction of the amendment to the NHI Act.

Hitherto, the question of how policy makers might raise health service consumers’ policy awareness or enforce policies to meet those consumers’ needs has seldom been examined with respect to medical management or public policy studies in Taiwan. Accordingly, the present study analyzes the difference between transparency of information disclosure and associated demands from the health service consumer’s perspective. To verify the intentions with mandatory information disclosure and enrich the local literature in this regard in Taiwan, this study also compares how health service providers and consumers are associated by different levels of such disclosure.

## 2. Materials and Methods

### 2.1. Study Design and Sampling

This study obtained research data using a questionnaire survey. We used a different questionnaire design for different research subjects. We aimed to test health service consumers’ concerns about the level of mandatory information disclosure by collecting data from such consumers in Taiwan aged over 20 years through telephone interviews. The interviews were conducted based on a telephone directory as the sampling list. We created computerized files using phone numbers found in the directory; for a stratified sampling of the telephone numbers, we divided the numbers into 23 stratifications. We sampled the numbers at random, and we replaced the last two digits of those numbers. Out of 1100 randomly chosen inhabitants were interviewed using computer-assisted telephone interviews (CATI). In that way, the numbers used in our analysis differed from those in the telephone directory and could be employed as samples for representative regional data, based on national demographics. We conducted the interviews between 6 p.m. and 10 p.m. on weekdays to prevent bias toward non-occupational groups. Taiwan’s population aged 20 years and above was 18,914,286. The CATI took place over 4 months from March to June 2013. In all, 413 questionnaires were returned, among which there were 384 effective questionnaires, with an overall valid response rate of 34.9%.

This research aimed to examine the transparency of information disclosure, which falls into the category of institutional constructs. Based on theories of institutional constructs, we designed a questionnaire targeting the 476 medical institutions listed as NHI contract hospitals in Taiwan in 2013. The research subjects were senior hospital executives, who received questionnaires by mail. Senior hospital executives were requested to complete the questionnaires anonymously. We also took two steps to verify the validity of the responses from the participants. To ensure that the respondent was an executive manager, we confirmed that fact with the subject by telephone and e-mail. To promote the motivation to make accurate responses, we also provided gifts to participants and promised to share with them the study results upon completion. In all, 201 valid questionnaires were returned and the return rate was 42.2% (Table 1). We conducted goodness-of-fit tests regarding the county or city of the hospital’s location and the hospital’s hierarchy. We found that the two characteristics showed no significant differences (*p* > 0.1). Thus, we could consider the samples representative. Non-response bias was examined using the extrapolation method [40], which compared early responses (March to April 2013) with late responses (May to June 2013) in terms of items surveyed in the sample. The test results showed that there was no bias included in the research since no significant differences were found at the 0.05 level.

### 2.2. Research Tools

The questionnaire we prepared includes scales for aspects of the respondents’ backgrounds and transparency of information disclosure. To develop the questionnaire, we built an original database based on domestic and foreign studies. We decided on the content of the first draft and then chose a way to measure the questionnaire scores. Finally, to complete the final form of the questionnaire, we modified it in terms of content validity and face validity; we made rough adjustments to the questions after conducting preliminary interviews. Thus, the questionnaire possessed specific validity with respect to concepts and measurement of variables. Of the 33 original questions in the utility scale, we revised five (Table A1 in Appendix A). Cronbach’s α in the hospitals’ overall reliability analysis was 0.891; Cronbach’s α in the health service consumers’ overall reliability analysis was 0.761. Thus, the evidence-based data in the present study had appropriate reliability.

### 2.3. Variable Measures, Reliability, and Validity

We based the scales we applied regarding transparent information disclosure on previous investigations [10,17]. In line with our study concept, we divided information disclosure into the following categories: disclosure of medical management; disclosure of medical treatment; disclosure of medical quality; disclosure of financial statements; disclosure of information about violations; and disclosure of insured bed ratio; and awareness and concerns. We used a Likert five-point scale as the data measurement standard. Possible answers were as follows: strongly agree; agree; neither agree nor disagree; disagree; and strongly disagree. The scales were subjected to a content validity test conducted by two specialists in medical management and six invited executive officers (two each from the medical center, regional hospital, and community hospital). The specialists’ average content validity index for the suitability of each question was 0.909. Before distributing the questionnaire, we submitted it to each hospital for approval and confirm whether it required modification. After modifying the questionnaire, we presented it to the institutional review board (IRB) of Show Chwan Memorial Hospital for review and approval (IRB No. 1020201). Thereafter, we used the questionnaire in testing.

We also conducted a construct validity analysis on our study concept by means of a confirmatory factor analysis; we categorized the analysis into two measuring systems based on the concept of split-half reliability. The result showed that in term of goodness-of-fit, the degree of freedom in a chi-square distribution was 2.334 (RMR = 0.06; GFI = 0.853; AGFI = 0.798; NFI = 0.830; IFI = 0.895; TLI = 0.870; and RMSEA = 0.08). In terms of convergent validity, the factor loadings for degree of use of external knowledge sources all attained statistical significance (*p* < 0.01). Moreover, the AVE values of the degree of use of the four external knowledge sources were all higher than the positive two-dimensional correlations after the same were radiated. The scales applied in this study apparently had good discriminant validity.

### 2.4. Statistical Analysis

We used SPSS for Windows, version 18.0, for data compilation and analysis and set the standard of significance at 0.05. First, the descriptive statistics related to the hospitals focused on the demographic attributes of the study subjects; we used the *t* test to explore differences in health service consumers’ awareness and concerns regarding transparency of hospital information disclosure. Second, the descriptive statistics relating to hospitals focused on hospital information and demographic characteristics of the study subjects. We used the chi-square test to compare the characteristics of medical centers, regional hospitals, and community hospitals in the samples. To prevent the chi-square test being affected by deficient samples, we conducted the test item by item with respect to categorical data analysis. If the expected cell number in some category in the variable analysis was less than 5, we used the stricter Fisher’s exact test. Finally, we used analysis of variance to examine differences in the awareness of health service providers and consumers regarding mandatory information disclosure.

## 3. Results

### 3.1. Sample Characteristics

We obtained 384 valid samples from consumers and 196 samples from health service providers (hospitals). Among the samples from health service consumers, females accounted for 59.3%; 34.9% were aged 30 years or under and 45.3% 31–50 years old. College graduate degrees or higher were held by 65.1%. Most respondents were engaged in service business (29.9%). We used the chi-square test to analyze the subjects by sex, and we found significant differences in terms of age and occupation (*p* < 0.05; Table 2). We undertook an additional analysis to check concerns over the small sample size. According to Dillman [41], sample size can be determined using the following equation:

$$N_{SAMPLE}=\frac{\left(N_{P}\right)\left(p\right)\left(1-p\right)}{\left(N_{P}-1\right){\left(E/C\right)}^{2}+\left(p\right)\left(1-p\right)}$$

where *N_SAMPLE_* signifies the sample size, *N_P_* the population size, *E* tolerance error, *C* the *Z* value (=1.96) of the 95% confidence interval, and *p*, 1 − *p* population variance. In the case of a normal distribution and fitting the expected characteristics, the maximum population variance is 50%. According to the above equation, the minimum sample size is 384. The number of our sample was 384; thus, our sample size was appropriate.

With respect to hospital level in the NHI program, the study subjects were occupied as follows: medical centers, 12 (return rate, 54.5%); regional hospitals, 45 (return rate, 54.8%), and community hospitals, 144 (return rate, 38.7%; Table 1). Regarding hospital ownership, the subjects responded as follows: public hospitals, 43 (21.4%); private hospitals, 112 (55.7%); and corporation hospitals, 46 (22.9%). According to location of branch offices of the National Health Insurance Bureau, the subjects responded as follows: hospitals in the Taipei area, 35 (17.4%); hospitals in North Taiwan, 24 (11.9%); hospitals in Central Taiwan, 50 (24.9%); hospitals in South Taiwan, 24 (11.9%); hospitals in the Kaohsiung and Pingtung areas, 58 (28.9%); and hospitals in East Taiwan, 10 (5%).

Among the interviewees, women accounted for 58.2%. The age of 46.8% of the interviewees was 41–50 years; 57.2% of interviewees had service seniority of 11 or more years; 50% or more had master’s or doctoral degrees; 25.8% were superintendents or vice-superintendents; 74.1% were senior hospital executive officers; and 76.1% were officers working in administrative departments. We categorized the interviewees’ data by medical center, regional hospital, and community hospital with respect to health insurance benefits. From the analysis conducted using the chi-square test, there were significant differences in ownership, level of education, and occupational position (*p* < 0.05) (Table 3).

### 3.2. Analysis of Transparency of Information Disclosure and Related Concerns

In term of transparency of hospital information disclosure and health service consumers’ related concerns, transparency of the medical management process was higher than the concerns (*p* < 0.01). In a similar fashion, we made the following findings: transparency of the medical treatment process was higher than concerns (*p* < 0.001); transparency of drug safety was higher than concerns (*p* < 0.01); transparency of medical quality was higher than concerns (*p* < 0.001); transparency of complaint channels and settlement results were lower than concerns (*p* < 0.01); transparency of patient satisfaction survey result disclosure was lower than concerns (*p* < 0.01); transparency of hospital accreditation result disclosure was higher than concerns (*p* > 0.1); transparency of financial statement disclosure was lower than concerns (*p* < 0.1); transparency of violation information disclosure was higher than concerns (*p* < 0.1); and transparency of insured bed ratio disclosure was higher than concerns (*p* < 0.05). Clearly, only three variables (complaint channel, patient satisfaction, and financial statement disclosure) showed concerns of being higher than transparency; the other variables showed the transparency of disclosure to be higher than concerns. The top three areas for transparency of information disclosure chosen by health service providers were drug safety disclosure, insured bed ratio disclosure, and hospital assessment result disclosure. The top three areas of consumer concern were patient satisfaction survey result disclosure, complaint channel and settlement result disclosure, and financial statement disclosure (Table 4).

In a comparison among health service providers and consumers regarding the transparency of hospital information disclosure, we identified the following variables: medical management; medical treatment; drug safety; medical quality; complaint channel and settlement result; patient satisfaction survey result; hospital assessment result; financial statement; violation information; and insured bed ratio. This result shows that the transparency considered by health service providers was higher than that regarded by health service consumers. The difference was significant (*p* < 0.001; Table 5).

## 4. Discussion

This study aimed to compare differences in the transparency of and demand for mandatory information disclosure for NHI in Taiwan. We examined hospitals and health service consumers there. We identified significant differences in the transparency of information disclosure as considered by health service consumers and their concerns about information disclosure. We found transparency of disclosure regarding such areas as medical management, medical treatment, drug safety, medical quality, hospital assessment result, violation information, and insured bed ratio to be higher than health service consumers’ concerns in those areas. Analysis of the transparency of information disclosure between health service providers and consumers also revealed some significant differences; this was primarily due to health service providers agreeing with the transparency but health service consumers disagreeing. We present the theoretical outcomes of our results in the following section.

In terms of hospital information disclosure legally required by the Taiwan government, health service consumers clearly do not have major concerns. This result is in keeping with the findings of a previous study [42]. That investigation showed that the groups with high concerns about hospital financial information disclosure were limited to the media, researchers, experts and scholars, the Taiwan Health Care Reform Foundation, and private certified public accountant firms. Further, the difference in health service providers’ and consumers’ awareness of disclosure probably resulted from the Second Generation NHI. That amendment allowed the public access to important information about hospital violations with a view to curbing hospitals and medical institutions that intended to break the rules, and it urged the public to co-supervise NHI-contracted medical institutions [10]. However, previous surveys and studies have found that the public had major concerns about available medical treatment information: 70% of participants indicated that they would review available information before seeking medical treatment; they would even consider assessment results to determine whether they should change their physicians [43]. This indicates that the public needs relevant medical treatment information in decision making. However, medical management is highly complex and uncertain. If medical management comes under intensive supervision [44,45], the physician’s professional autonomy will be directly impaired [46,47]. Therefore, health service consumers may consider the information disclosure to be insufficient.

Moreover, information disclosure about medical quality encompasses details of care quality and the performance standards of institutions with respect to availability, expertise, and interpersonal communication [48]. Thus, medical quality information disclosure is expected to result in a “survival of the fittest” situation in term of suppliers [48,49]. To achieve information disclosure about medical quality related to health insurance, relevant, accurate information has to be made available to the public in a form it understands. If the public cannot understand medical quality information, it might consider such information useless [50]. Therefore, when providing the public with medical quality information, it is necessary to reduce the burden for the public in dealing with such information [50]. It also essential to use information technology when disclosing information [51,52] so as to increase its applicability. Our study is an association study, and the results should not be interpreted causally, a selection bias is likely to exist because of the transparent purpose of the study, and the relatively low response rate to our survey may limit the generalizability of our findings. Furthermore, a possible response bias is another limitation.

## 5. Study Limitations

This study has limitations. For example, we examined the transparency of information disclosure in terms of operational variables, validity content, time, and institutional norms. Further research should address the future of hospital governance, and the results of such studies should be based on a theoretical framework of mutual control.

## 6. Conclusions

As a whole, the concepts referred to in this paper contribute to the theoretical framework for mandatory information disclosure. The important findings, theoretical meanings, and policy suggestions we have made with respect to the study results may be stated as follows.

(1) The findings include: (I) Health service consumers are not greatly concerned about mandatory information disclosure. They are more concerned about complaint channels and settlement results, patient satisfaction survey results, and disclosures of hospital financial statements; (II) Some significant differences exist between health service providers’ and consumers’ awareness of the transparency of information disclosure.

(2) Policy suggestions: It may not be possible for a government to properly interpret the information provided by a hospital. Therefore, when a hospital discloses information, it is necessary for the government to consider the information’s applicability. Toward improving medical expertise and information asymmetry, the government has to reduce the burden among health service consumers in dealing with this information, and it has to use the information effectively.

## Figures and Tables

**Table 1:** Hospital level of national health insurance (NHI) program (*n* = 201).

Variable	All Hospitals *n* = 476	Questionnaire Returned by Hospitals *n* = 201	Return Rate 42.2%
Medical center	22	12	54.5%
Regional hospital	82	45	54.8%
Community hospital	372	144	38.7%

**Table 2:** Health service consumer characteristics (*n* = 384).

Variable		All	%	Male	%	Female	%	χ^2^(df)	*ρ*
**Age (years)**								11.73(3)	0.008
	<30	134	34.9	40	10.4	94	24.5		
	31–40	85	22.1	38	9.9	47	12.2		
	41–50	89	23.2	38	9.9	51	13.3		
	>50	76	19.8	40	10.4	36	9.4		
**Education level**								2.85(4)	0.582
	Graduate School	30	7.8	15	3.9	15	3.9		
	University	250	65.1	97	25.3	153	39.8		
	Senior High School	82	21.4	37	9.6	45	11.7		
	Junior High School	12	3.1	4	1.0	8	2.1		
	Primary School	10	2.6	3	0.8	7	1.8		
**Occupation**								21.69(4)	0.001
	Student	105	27.3	25	6.5	80	20.8		
	Military, Civil and Teaching Staff	28	7.3	15	3.9	13	3.4		
	Business	115	29.9	48	12.5	67	17.4		
	Other	98	25.5	53	13.8	45	11.7		
	No	38	9.9	15	3.9	23	6.0		

**Table 3:** Health service provider characteristics (*n* = 201).

Variable		All	%	Medical Center	%	Regional Hospital	%	Community Hospital	%	χ^2^(df)	*ρ*
**Hospitals ownership**										67.3(4)	0.001
	Public hospital	43	21.4	2	1.0	20	10.0	21	10.4		
	Private hospital	112	55.7	2	1.0	5.	2.5	105	52.2		
	Non-profit proprietary	46	22.9	8	4.0	20	10.0	18	9.0		
**BNHI branch**										8.2(10)	0.602
	Taipei	35	17.4	2	1.0	8	4.0	25	12.4		
	Northern	24	11.9	1	0.5	6	3.0	17	8.5		
	Central	50	24.9	3	1.5	8	4.0	39	19.4		
	Southern	24	11.9	3	1.5	9	4.5	12	6.0		
	Kaohsiung & Pingtung	58	28.9	2	1.0	12	6.0	44	21.9		
	East	10	5.0	1	0.5	2	1.0	7	3.5		
**Gender**										0.598(2)	0.741
	Male	84	41.8	6	3.0	20	12.4	58	28.9		
	Female	117	58.2	6	3.0	25	10.0	86	42.8		
**Age (years)**										10.17(6)	0.253
	<30	7	3.5	0	0.0	2	1.0	5	2.5		
	31–40	37	18.4	1	0.5	5	2.5	31	15.4		
	41–50	94	46.8	7	3.5	27	13.4	60	29.9		
	>50	63	31.3	4	2.0	11	5.5	48	23.9		
**Years of Service**										9.71(6)	0.137
	<10	86	42.8	7	3.5	23	11.4	67	33.3		
	11–15	49	24.4	5	2.5	17	8.5	35	17.4		
	16–20	42	20.9	5	2.5	12	6.0	26	12.9		
	>21	24	11.9	7	3.5	3	1.5	16	8.0		
**Education level**										43.95(6)	0.001
	Graduate School-Doctoral	12	6.0	0	0.0	6	3.0	6	3.0		
	Graduate School-Masters	89	44.3	9	4.5	34	16.9	46	22.9		
	University	65	32.3	2	1.0	4	2.0	59	29.4		
	Senior High School	35	17.4	1	0.5	1	0.5	33	16.4		
**Departments**										8.12(4)	0.087
	Medical	32	15.9	0	0.0	6	3.0	26	12.9		
	Nursing	16	8.0	0	0.0	1	0.5	15	7.5		
	Administrative	153	76.1	12	6.0	38	18.9	103	51.2		
**Occupational position**										10.26(4)	0.036
	Superintendents	26	12.9	0	0.0	2	1.0	24	11.9		
	Vice Superintendents	26	12.9	0	0.0	9	4.5	17	8.5		
	Chief Executive Officer	149	74.1	12	6.0	34	16.9	103	51.2		

**Table 4:** Analysis of transparency of mandatory information disclosure and concerns of health service consumers (*n* = 384).

Variable	Transparency		Concern		*t*	*ρ*
	Mean	SD	Mean	SD		
Medical management process	3.18	0.80	2.46	0.76	12.035	0.003
Medical treatment process	3.35	0.79	2.40	0.78	15.268	0.001
Drug safety	3.36	0.84	2.23	0.83	17.529	0.003
Medical quality	3.20	0.80	2.30	0.94	13.138	0.001
Complaining channel and settlement result	3.10	0.87	3.33	0.73	–3.454	0.001
Patient satisfaction survey result	3.11	0.81	3.37	0.67	–4.213	0.001
Hospital accreditation	3.20	0.91	2.50	0.93	10.339	0.239
Financial statement	2.73	0.85	2.83	0.81	–0.874	0.063
Violation information	2.77	0.86	2.44	0.86	5.620	0.081
Insured bed ratio	3.02	0.96	2.37	0.83	10.596	0.050

**Table 5:** Analysis of variance of mandatory information disclosure.

Variable	Providers (*n* = 201)		Consumers (*n* = 384)		*F*	*ρ*
	Mean	SD	Mean	SD		
Medical management process	3.68	0.07	3.18	0.80	54.089	0.001
Medical treatment process	3.94	0.66	3.35	0.79	82.884	0.001
Drug safety	4.02	0.60	3.36	0.84	99.821	0.001
Medical quality	3.87	0.71	3.20	0.80	97.530	0.001
Complaining channel and settlement result	3.85	0.71	3.10	0.87	107.175	0.001
Patient satisfaction survey result	3.70	0.84	3.11	0.81	68.146	0.001
Hospital accreditation	4.21	0.70	3.20	0.91	192.038	0.001
Financial statement	3.04	1.10	2.73	0.85	10.557	0.001
Violation information	3.56	0.89	2.77	0.86	106.693	0.001
Insured bed ratio	4.37	0.68	3.02	0.96	314.317	0.001

## Appendix A

**Table A1:** Questionnaires.

Questionnaires of health services providers (10 major items, 30 questions) 1. The hospital medical management process disclosure—disease treatment process 2. The hospital quality of medical treatment interactive disclosure—medical treatment process 3. The hospital health information disclosure—drug safety 4. The hospital medical quality indicators information disclosure 5. The hospital complaining channel and settlement result disclosure 6. The hospital patient satisfaction survey results disclosure 7. The hospital accreditation results disclosure 8. The hospital financial statements active online disclosure 9. The hospital violation information disclosed 10. The hospital insurance bed ratio disclosure
Questionnaires of health services consumers (10 major items, 30 questions) 11. The hospital medical management process concern—disease treatment process 12. The hospital quality of medical treatment interactive concern—medical treatment process 13. The hospital health information concern—drug safety 14. The hospital medical quality indicators information concern 15. The hospital complaining channel and settlement result concern 16. The hospital patient satisfaction survey results concern 17. The hospital accreditation results concern 18. The hospital financial statements active online concern 19. The hospital violation information concern 20. The hospital insurance bed ratio concern
